# Conserved Genes in Highly Regenerative Metazoans Are Associated with Planarian Regeneration

**DOI:** 10.1093/gbe/evae082

**Published:** 2024-04-23

**Authors:** Shankar C R R Chereddy, Takashi Makino

**Affiliations:** Graduate School of Life Sciences, Tohoku University, Sendai 980-8578, Japan; Graduate School of Life Sciences, Tohoku University, Sendai 980-8578, Japan

**Keywords:** comparative genomics, regeneration, planarian

## Abstract

Metazoan species depict a wide spectrum of regeneration ability which calls into question the evolutionary origins of the underlying processes. Since species with high regeneration ability are widely distributed throughout metazoans, there is a possibility that the metazoan ancestor had an underlying common molecular mechanism. Early metazoans like sponges possess high regenerative ability, but, due to the large differences they have with Cnidaria and Bilateria regarding symmetry and neuronal systems, it can be inferred that this regenerative ability is different. We hypothesized that the last common ancestor of Cnidaria and Bilateria possessed remarkable regenerative ability which was lost during evolution. We separated Cnidaria and Bilateria into three classes possessing whole-body regenerating, high regenerative ability, and low regenerative ability. Using a multiway BLAST and gene phylogeny approach, we identified genes conserved in whole-body regenerating species and lost in low regenerative ability species and labeled them Cnidaria and Bilaterian regeneration genes. Through transcription factor analysis, we identified that Cnidaria and Bilaterian regeneration genes were associated with an overabundance of homeodomain regulatory elements. RNA interference of Cnidaria and Bilaterian regeneration genes resulted in loss of regeneration phenotype for *HRJDa*, *HRJDb*, *DUF21*, *DISP3*, and *ARMR* genes. We observed that *DUF21* knockdown was highly lethal in the early stages of regeneration indicating a potential role in wound response. Also, *HRJDa*, *HRJDb*, *DISP3*, and *ARMR* knockdown showed loss of regeneration phenotype after second amputation. The results strongly correlate with their respective RNA-seq profiles. We propose that Cnidaria and Bilaterian regeneration genes play a major role in regeneration across highly regenerative Cnidaria and Bilateria.

**Significance**

Regeneration by design is an extension of developmental processes where regenerative processes reactivate specific developmental processes in a context-dependent mechanism. This critical process varies widely across metazoan species and is largely lost in Bilaterians. Here, we classified regeneration across Cnidarian and Bilaterian lineages and identified a set of genes that are evolutionarily conserved in highly regenerative species across the lineages. We show that these genes could potentially be part of developmental pathways and provide evidence for their importance during regeneration. These findings offer insights into previously unidentified regeneration-related genes, potentially preserved across vast evolutionary timescales of the Cnidarian and Bilaterian lineages and could hold the key to unlocking the secrets of regeneration and its variability.

## Introduction

Regeneration and its differences across Metazoa have been a scientific conundrum that is yet to be fully understood. For example, axolotls (Phylum Chordata, Class Amphibia, Order Urodela) can regenerate their limbs post amputation, i.e. appendage regeneration. Despite being an inherently complex mechanism that replaces lost tissue and bone structures, this regenerative ability can be considered low regenerative ability (LRA) since it is limited to appendages and organs. However, lancelets (Phylum Chordata, Subphylum Cephalochordata) can regenerate their trunk regions, i.e. anterior and posterior regions (Somorjai et al. 2012). This mode of trunk regeneration presents a higher order of regeneration compared with appendage regeneration in axolotls and can be considered high regenerative ability (HRA). Due to the lack of trunk regeneration, we exclude species like axolotls and salamanders from the HRA group and include them in the LRA group. In addition, species like the planaria and hydra show a higher order of regeneration as they can regenerate entire body structures from minute tissue fragments (Alvarado 2000; Bely and Nyberg 2010). These differences in regeneration were classified across metazoan species in Bely and Nyberg (2010).

Despite the high variability of regeneration, there are biological processes that are common across various species. For example, Wnt signaling has been shown to be crucial for anterior and posterior regeneration in planaria (Kobayashi et al. 2007; De Robertis 2010). Wnt signaling has been shown to be important in axial formation in hydra (Hobmayer et al. 2000; Tursch et al. 2022). Wnt signaling has also been implicated in tail regeneration in amphioxus and limb regeneration in axolotls (Liang et al. 2019; Lovely et al. 2022). The underlying mechanism of Wnt signaling pathways indicates a potentially conserved regenerative mechanism across regenerative metazoan species.

**Fig. 1.**
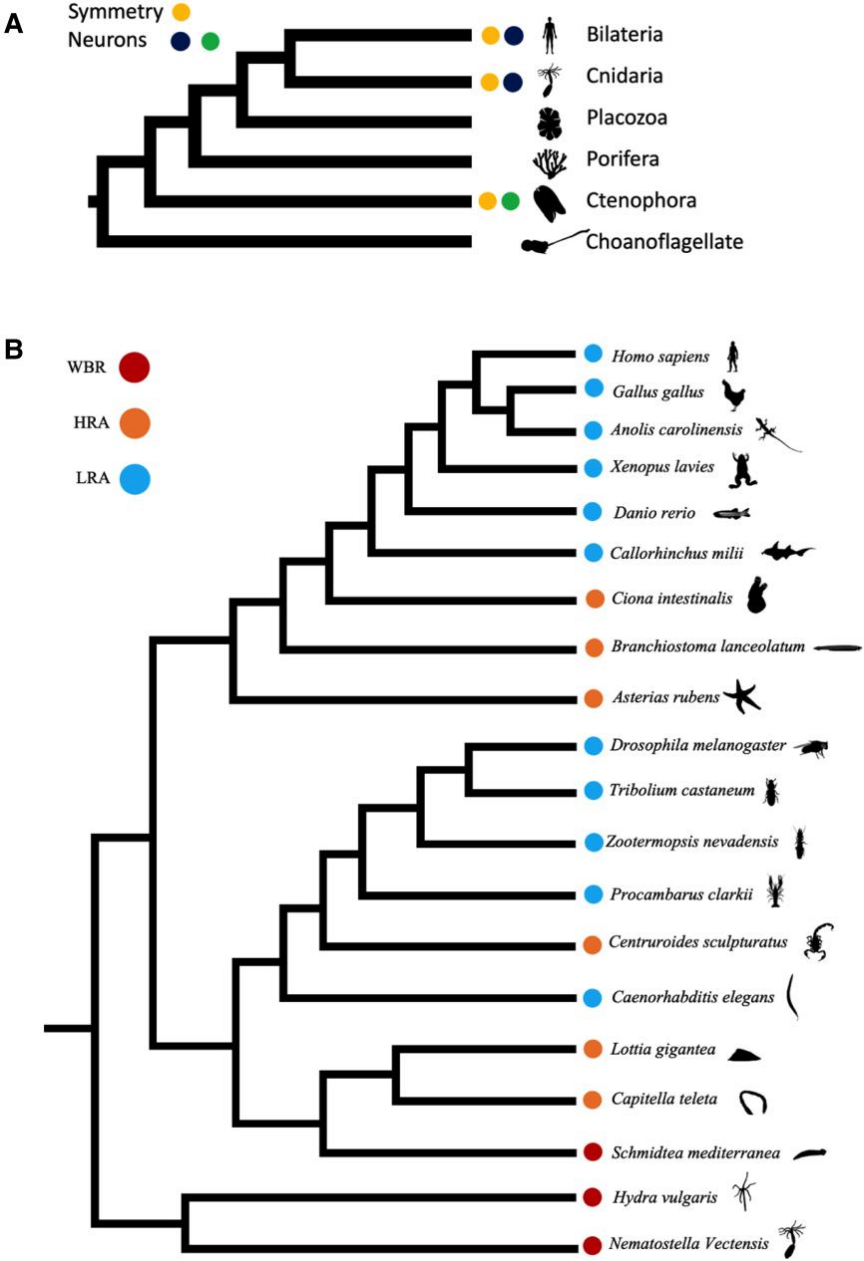
Reclassification of regenerative species. a) Early metazoans, Cnidaria, and Bilateria were classified according to the presence of symmetry and neuronal systems. b) Cnidarians and Bilaterians were classified into three groups: WBR, HRA, and LRA. Multiway mutual BLAST hits across HRA species were contrasted with LRA species to identify genes conserved across HRA species and lost in evolution across LRA species. Silhouettes were obtained from PhyloPic (http://phylopic.org/).

Previously, Cao et al. (2019) used the differences in regeneration across metazoan and classified species into high regenerative and low regenerative species. This was used to identify a set of genes conserved across highly regenerative metazoans aptly termed highly regenerative species-specific JmjC domain-encoding genes (*HRJD*). However, one of the drawbacks of the previous research article was the lack of specificity involved in selecting candidate species to identify highly regenerative species-specific genes. In this study, we modified the previous classification to exclude early metazoans from the highly regenerative species group. Our reasoning was based on two main factors involving the presence of symmetry and neuronal systems as indicated in Fig. 1A. Among the early metazoan species of Ctenophora, Porifera, and Placozoa, only Ctenophores are known to possess both symmetry and a neuronal system (Dunn et al. 2015; Moroz and Romanova 2022). Both Porifera and Placozoa possess no form of symmetry and no known neuronal cells. The symmetry of Ctenophores is also limited to rotational symmetry and markedly differs from the radial and bilateral symmetry observed in Cnidarians and Bilaterians, respectively (Finnerty 2003; Manuel 2009). Also, Moroz and Kohn (2016) suggest the potential independent emergence of neuronal systems in Ctenophore and junction of Cnidarians and Bilaterians. Cnidarian neuronal systems are also different compared with the Ctenophore counterparts (Galliot et al. 2009; Watanabe et al. 2009). Based on this data regarding symmetry and neuronal systems, we hypothesized that the regeneration observed in early metazoans could be different to the regeneration observed in Cnidarians and Bilaterians. The emergence of neuronal systems and symmetry at the node of Cnidarians and Bilaterians could indicate a common lineage for the regeneration mechanisms observed in these phyla. We hypothesized that the last common ancestor of Cnidaria and Bilateria possessed HRA which was eventually lost as complexity increased in the clade. To test our hypothesis, we identified conserved orthologs unique to highly regenerative species and verified their effect on regeneration on model planarian. We present evidence through gene phylogenetic trees of orthologs unique to highly regenerative species and verify their potential function through RNA interference (RNAi) experiments (Shibata and Agata 2018).

## Results

### Conserved Orthologs in High Regenerative Species

In total, we identified 25 orthologous groups conserved in WBR species (supplementary table S4, Supplementary Material online). Multiway BLAST and National Center for Biotechnology Information (NCBI) database screening revealed that only ten of these genes were uniquely conserved in highly regenerative species, which included the previously characterized gene *HRJD* (Table 1; Cao et al. 2019). *ARMR*, *MFS*, *SLR1101*, and *TLR* were only conserved in WBR/HRA species. The gene trees of *HRJD*, *CAPED*, *AVT3C*, *DUF21*, and *AUL36* showed distinct clusters for the orthologs in high regenerative species and the corresponding paralogs in low regenerative species (supplementary fig. S3, Supplementary Material online). *DISP3* also showed distinct clusters; however, we noticed that *DISP3* was lost in both *Asteria rubens* and *Ciona intestinalis*. But we found that *DISP3* was conserved in Mollusca and Annelida; these groups were previously identified as high regenerative groups in Cao et al. (2019).

**Table 1:** Orthologous genes conserved in WBR species after second screening

Gene	Description
*HRJDa & HRJDb*	High regenerative species-specific JmjC domain-encoding gene
*SLR1101*	Universal stress protein
*CAPED*	Cysteine-rich secretory protein
*AUL36*	Ankyrin repeat-rich protein
*TLR*	Toll-like receptor
*MFS*	Major facilitator superfamily
*AVT3C*	Solute carrier family
*ARMR*	Armadillo repeat-rich protein
*DISP3*	Protein dispatched homolog
*DUF21*	Uncharacterized transmembrane protein

### Biased Distribution of Transcription Factor Binding Sites in Upstream Region of Cnidaria and Bilaterian Regeneration Genes

**Fig. 2.**
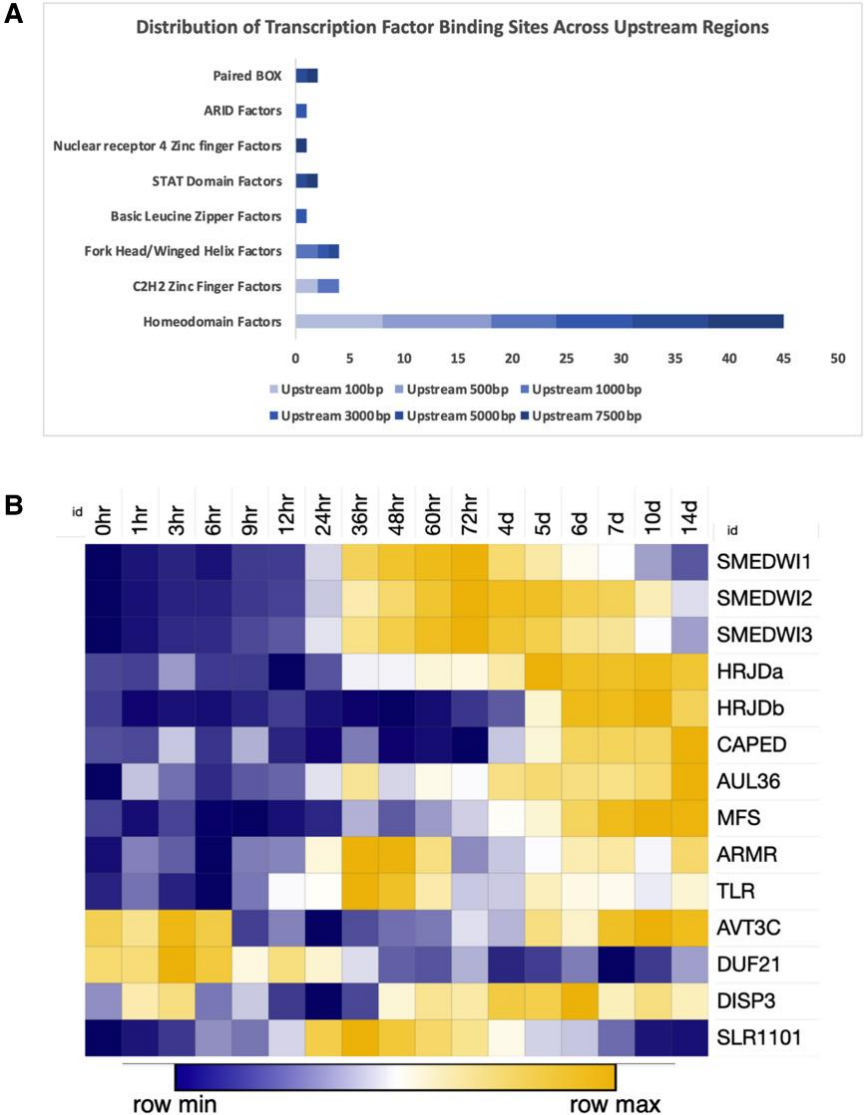
Characterization of CBR genes. a) Expression profile of all CBR genes. Expression values used are FPKM values. *SMEDWI1*, *SMEDWI2*, and *SMEDWI3* were included as contrast. b) TF domain enrichment across different upstream sequence sizes. TFBSs were identified in upstream sequences, and the corresponding transcription factor domains were tallied and plotted on the *x* axis. Heatmap was generated using Morpheus (https://software.broadinstitute.org/morpheus/).

To ascertain the role of Cnidaria and Bilaterian regeneration (CBR) genes, we tried to decipher the potential regulatory elements involved. For this purpose, we used CiiiDER to identify transcription factor binding sites (TFBSs) in upstream regions of CBR genes for *Schmidtea mediterranea*, *Dugesia japonica*, *Nematostella vectensis*, *Hydra vulgaris*, and *C. intestinalis*. We observed that the upstream regions in general are rich in homeodomain TFBS (Fig. 2A). This was consistent across all CBR genes and was also observed in individual CBR upstream TFBS analysis. We observed that C2H2 zinc finger factors and fork head/winged helix TFBSs were also enriched in the upstream region. However, homeodomain TFBS were overwhelmingly enriched compared with all other TF classes. This could indicate that these genes are potentially controlled by homeodomain recognizing transcription factors (TFs) which have been previously found to be involved extensively in developmental processes (Banerjee-Basu and Baxevanis 2001). The important distinction here is that we do not segregate homeodomain factors into the various families like HD-LIM, paired related factors, or HOX factors and only broadly recognize the enrichment of the homeodomain TFBS itself.

### Expression Patterns for CBR Orthologs in the Regeneration Process

To explore the RNA expression profile of CBR genes during regeneration, we examined expression data sets of CBR orthologs in *S. mediterranea* during regeneration process to identify specific patterns of expression (NCBI GEO: GSE107875). *SMEDWI1*, *SMEDWI2*, and *SMEDWI3* were used for contrast purposes due to their critical role in stem cell maintenance and homeostasis (Reddien et al. 2005, Palakodeti et al. 2008). In Fig. 2B, *SMEDWI* expression changed 24 h post amputation, and we observed that *HRJDa*, *AUL36*, *ARMR*, and *DISP3* showed similar expression patterns. Based on Gentile et al. (2011), this can be considered to coincide with the stem cell proliferative and differentiation phases of planarian regeneration. It is to be noted that *DISP3* did show expression changes in the early regeneration stages before 6 h post amputation. This could indicate a potential role in the wound response phase of planarian regeneration. *SLR1101* also showed a similar expression change around 24 h post amputation, but this change was arrested after 4 d post amputation. *HRJDb*, *CAPED*, and *MFS* were primarily expressed in the latter stages of regeneration indicating a potential role in the stem cell differentiation phase of planarian regeneration. *AVT3C* and *DUF21* showed expression changes immediately post amputation indicating a role in the wound response phase of planarian regeneration.

### Knockdown Phenotype after RNAi

**Fig. 3.**
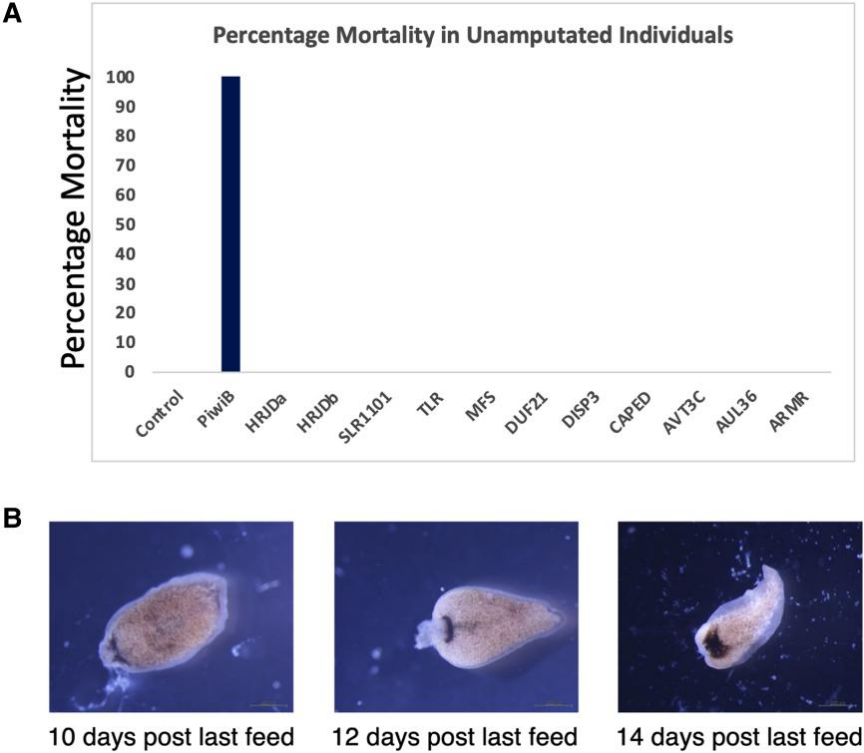
RNAi mortality and phenotype in unamputated individuals. a) Percentage mortality in unamputated planaria treated with dsRNA (*N* = 10). The *y* axis denotes percent mortality. b) HRJDa dsRNA treatment after 10, 12, and 14 d post last feeding showing loss of head phenotype.

We used RNAi to decipher the potential role of CBR genes in planarians (*Dugesia* sp.). We conducted two distinct experiments using RNAi to identify the function of CBR genes. The first experiment was conducted without any amputation to observe any potential phenotype in response to gene knockdown. *DjPiwiB* was used as positive control (Hayashi et al. 2010; Shibata et al. 2016). We observed that all planaria in the *DjpiwiB* condition perished within 1 week after the last feeding (Fig. 3A). In contrast, we did not observe any adverse phenotypes in any of the other treatments except *HRJDa*. We observed that *HRJDa*-treated individuals started to lose their heads around 10 d after the last feed and completely lost their head by 14 d after last feed (Fig. 3B). The planaria did not self-amputate to lose these heads but instead lysed the tissue in the anterior region to reach a head-less state.

**Fig. 4.**
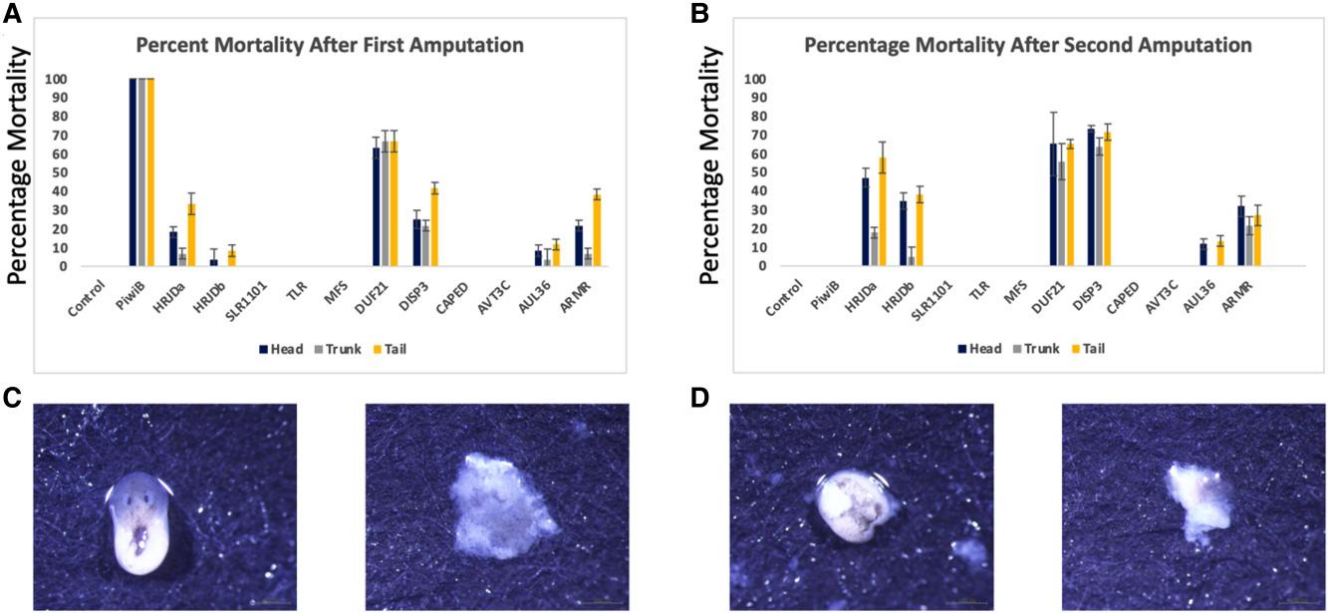
RNAi mortality and phenotype in amputated individuals. a) and b) Percentage mortality observed after first (*N* = 20 each for head, trunk, and tail) and second amputation (*N* = surviving fragments post first amputation), respectively. The *y* axis denotes the percent mortality. Error bars generated using standard deviation of multiple experiments. c) and d) Mortality phenotype observed in regenerating heads and tails, respectively.

We also observed RNAi phenotype in amputated individuals (first amputation; Fig. 4A). Control planaria treated with *DjpiwiB* died within 72 h post first amputation. High percentage mortality was observed in *DUF21* within 48 h post amputation. This phenotype corresponded with the expression pattern induced early after amputation (Fig. 2B). *DISP3* another gene with increase in expression immediately post amputation also showed high percent mortality during the early regeneration stages. *HRJDa*, *HRJDb*, and *ARMR* showed mortality starting 4 d post amputation albeit lower compared with *DUF21* and *DISP3*. We further amputated the surviving individuals again to observe if there was any change in mortality rates. This was to account for any potential phenotype that could be observed after multiple amputations (supplementary table S3, Supplementary Material online). We observed that *HRJDa* and *HRJDb* showed a higher percent mortality starting 4 d post amputation after the second amputation, and the results were in accordance with our previous findings in Cao et al. (2019). In addition, we observed an increase in mortality in *DISP3*-treated individuals starting at 24 h post second amputation. *DUF21* showed similar mortality rates in both the first and second amputations. *ARMR* also showed an increase in mortality after the second amputation indicating a role in the regeneration time line of planaria. In general, we observed that head and tail fragments showed a higher mortality rate potentially alluding to the lower amount of neoblasts present in these regions compared with the trunk region (Orii et al. 2005). We observed that most planaria that died in the early stages of regeneration died with the lysis phenotype with partial blastemas. Planarias that died during the later stages of regeneration showed an indication of being unwell following which they showed the lysis phenotype (Fig. 4C and D).

## Discussion

We found that ten HRA genes are conserved in highly regenerative Cnidaria and Bilateria. Although not all these genes were conserved in our HRA group, we believe that it showcases a marked difference in the regenerative landscape of Bilateria. As it is evident that the regeneration of Cnidaria and planaria represents the pinnacle of regeneration, i.e. whole-body regeneration (WBR), we believe the partial conservation of CBR genes in Echinoderms, Ascidians, and Cephalochordates is expected. Additionally, we observed that our CBR genes were also conserved in Chelicerata as previously observed with *HRJD* in Cao et al. (2019), although *HRJD* was not conserved in other athropods (Diptera, Coleoptera, Dictyoptera, and Decapoda). This led us to explore the regulatory regions of these genes, and the corresponding upstream regions of these CBR genes were enriched for motifs related to homeodomain and fork head/winged helix TFs. Homeodomain TFs have been studied extensively throughout metazoa and are known to play an extensive role throughout development by determining the body plan and controlling the pattern formation (Banerjee-Basu and Baxevanis 2001). A previous study has shown that Homeodomain TFs influence the early embryonic development of *Drosophila melanogaster* (Corsetti et al. 1992). Homeodomains have also been studied in planarian species and have been found to express along the anterior and posterior axis (Orii et al. 1999; Currie et al. 2016; Arnold et al. 2021) and shown to be crucial in the patterning of said axis during regeneration (Stornaiuolo et al. 1998). Fork head/winged helix TFs have previously been identified to be crucial in anterior pole regeneration (Scimone et al. 2014). It has been shown that C2H2 zinc finger factors play a role in neoblast maintenance and homeostasis (Lee 2023). These reports on TFs related to development and regeneration suggest that HRA genes could be involved in the molecular mechanisms of the regenerative process. Although our research does not specify which homeodomain factors are involved in regulating the upstream regions of CBR genes, we believe it provides a base for future studies designed around transcription factor complex formation.

Our RNAi experiments confirmed the previous findings in Cao et al. (2019) regarding *HRJDa* and *HRJDb* while also identifying a new loss of head phenotype for *HRJDa* without amputation. This result was in stark contrast to the results described in Cao et al. (2019). We believe this is due to the experiment ending prematurely at 7 d and the lower amount of dsRNA used for the feeding method. This result indicates that *HRJDa* could play a role in homeostasis and wound response aspects of the regenerative framework of planaria. The loss of head phenotype was previously observed in Cao et al. (2020). Based on previous research into head regeneration in planaria, it can be inferred that *HRJDa* could be related to maintenance of anterior body segments in planaria (Gurley et al. 2008; Tian et al. 2021; Wang et al. 2021). Also, demethylases have been implicated in numerous studies to modulate Wnt signaling pathways (Jiang et al. 2013; Lu et al. 2015; Lađinović et al. 2020). This could indicate a relationship with developmental and wound response processes, regulated via Wnt signaling pathways.

*DUF21* showed the most severe phenotype in terms of mortality within the first 48 h post amputation. *DUF21* also showed mortality in the trunk parts which was rarely observed in the *HRJD* genes. This phenotype is like that observed in *DjpiwiB* knockdown planarians where the mortality is observed consistently across trunk and nontrunk parts (Reddien et al. 2005; Palakodeti et al. 2008). The *DUF21* domain is a transmembrane region with unknown function (Yang et al. 2005; de Baaij et al. 2012). Despite the lack of information on the true function of *DUF21*, the domain has been found to be conserved across eukaryotes and prokaryotes. It is observed to occur with the cystathionine-beta-synthase (CBS) domains which have been implicated in metal ion homeostasis and signal transduction (Hao et al. 2016; Hao et al. 2021; Funato and Miki 2022). In another research study, upregulation of CBS domain containing proteins coincided with upregulation of multiple cancer-related factors and Wnt signaling pathway genes (Ascenção and Szabo 2022). Based on the regenerative processes discussed in Gentile et al. (2011), it can be inferred that *DUF21* is associated with either wound response or proliferative response-related genes.

*DISP3* also showed mortality during the first amputation which was further amplified after the second amputation. Like *DUF21*, *DISP3* also showed mortality in trunk parts of the planarian. The phenotype of both *DUF21* and *DISP3* was lysis phenotype which was very similar to *DjpiwiB* phenotype. Dispatched homolog 3 has been previously shown to regulate the release of hedgehog signaling proteins (Briscoe and Thérond 2013). Other dispatched homologs have been shown to enhance hedgehog signaling through cholesterol modifications (Cohen 2010; Tukachinsky et al. 2012). It is important to note that studies on colon cancer have identified that hedgehog signaling and Wnt signaling pathways are interlinked and implicate hedgehog signaling as a potential positive regulator (Song et al. 2015; Regan et al. 2017). Another study conducted in mice showed that *DISP3* controlled the fate of neural stem cell lineages and overexpression of *DISP3* was directly correlated to hyperproliferation in neural stem cells (Konířová et al. 2017). Like *DISP3*, *ARMR* also showed mortality in both first and second amputations; however, *ARMR* showed comparatively less trunk mortality. InterPro results in supplementary fig. S2K, Supplementary Material online, reveal the presence of armadillo repeats in protein sequence. Armadillo repeats are considered extremely versatile due to their protein structure (Tewari et al. 2010). They are involved in a variety of functions ranging from protein–protein interaction to signal transduction and are critical in maintaining signaling pathways (Coates 2003). Armadillo repeats have also been studied in planaria through *β-catenin* which comprises of multiple such repeats. *β-catenin* has been extensively studied in planaria and has been shown to control regeneration of anterior and posterior segments of planaria (Gurley et al. 2008; Umesono et al. 2013; Pascual-Carreras et al. 2023). This could indicate a potential relation to the Wnt signaling pathway that is at the center of numerous developmental processes and implicate it in wound response-related processes. The delayed response of *DISP3* and *ARMR* points to an association with stem cell differentiation which is usually observed in the latter stages of regeneration post blastema formation (Gentile et al. 2011).

## Conclusion

We identified a set of genes specifically conserved in highly regenerative metazoans. We have shown through RNAi experiments that some of these CBR genes are potentially associated with HRA in planarians. We propose that these genes could have been part of the ancestral genome of the last common ancestor of Cnidaria and Bilateria. We propose that the CBR genes lost in LRA species are associated with the loss of HRA. The findings in this study provide a platform to further advance our understanding of HRA through the largely unexplored HRA genes.

## Materials and Methods

### Identification of Conserved Orthologs in Highly Regenerative Species

To identify conserved orthologs, we segregated Cnidarian and Bilaterian species into three groups which are WBR species, HRA species, and LRA species (Fig. 1B) (Bely and Nyberg 2010). We included *N. vectensis*, *H. vulgaris*, and *S. mediterranea* in our WBR group and *C. intestinalis*, *Branchiostoma lanceolatum*, *A. rubens*, *Lottia gigantea*, and *Capitella teleta* in our HRA species. Protein sequences for *N. vectensis* and *B. lanceolatum* were obtained from NCBI genome (https://www.ncbi.nlm.nih.gov/genome/), and *A. rubens*, *C. intestinalis*, and *H. vulgaris* proteomes were obtained from Ensembl metazoan (release 57). *S. mediterranea* SchMedS2-high confidence proteome was obtained from Planmine (https://planmine.mpinat.mpg.de/). Proteomes for *Homo sapiens*, *Gallus gallus*, *Anolis carolinensis*, *Xenopus laevis*, *Danio rerio*, *Callorhinchus milii*, *D. melanogaster*, and *Caenorhabditis elegans* were downloaded from Ensembl (release 110), and those for *Tribolium castaneum*, *Zootermopsis nevadensis*, and *Procambarus clarkii* were obtained from Ensembl metazoan (release 57) in our LRA group. To identify conserved orthologs, a multiway mutual BLASTP was performed across all WBR and LRA species (threshold: *E*-value < 1e^−4^). We used the longest protein sequence for genes with alternatively spliced forms for the multiway BLASTP. Orthologous groups uniquely conserved in WBR species and lost in LRA species were identified as candidates associated with WBR ability. These genes were termed Cnidaria and Bilaterian ancestral genes (CBR). To clarify the uniqueness of the identified CBR genes, we confirmed that BLASTP hits were not found for whole amino acid sequences of any LRA species (arthropods, nematodes, and vertebrates) in NCBI database. This was to ensure that no LRA species had conserved any of the CBR genes while also accounting for within species variability of regenerative LRA species like axolotls and salamanders. We also used additional species to assay interspecies variability to confirm that the CBR genes are conserved in the hypothesized pattern based on their regenerative ability. We also identified orthologs for the CBR candidate genes in HRA species by a mutual BLASTP search (*N. vectensis* queries against protein sequences from HRA species; threshold: BLAST score > 100). When there were homologs of CBR genes in human as representative LRA species, we collected orthologs in LRA species for the human gene, and phylogenetic analysis was performed to investigate whether they were orthologs or paralogs for CBR. We used MAFFT to generate alignments which were used with RAxML to infer phylogenetic trees (Stamatakis 2014). To assess the inferred topology, parametric bootstrapping was performed with 100 replicates. Bootstrap values were calculated for each node of the maximum likelihood tree, and the resulting phylogenetic trees with corresponding bootstrap values are shown in supplementary fig. S3, Supplementary Material online. All genes were named based on their gene descriptions or domain structures. Due to our past research in *HRJD*, we chose to include both the paralogs *HRJDa* and *HRJDb* in our current study.

### TFBSs in Upstream Regions of CBR Candidate Genes

Candidate genes unique to CBR species were characterized by identifying TFBSs in upstream regions of the transcription start sites for all 14 genes. Upstream regions were divided into multiple levels of 100, 500, 1,000, 3,000, 5,000, and 7,500 bp. We used CiiiDER (Gearing et al. 2019) to identify over and underrepresented TF sites in these upstream sequences. Briefly, upstream regions of all genes in the genome were used to produce background gene list which was then used to contrast with the CBR gene list at the appropriate length of upstream sequence used. This resulted in giving a list of transcription factor sites that are uniquely enriched in upstream regions of CBR genes compared with the distribution across the background gene list. The JASPAR nonredundant vertebrate database was used to identify potential TFBSs across the upstream sequences (Sandelin et al. 2004; Castro-Mondragon et al. 2022). TFBSs enriched at the top 10 significance level and highest log2 enrichment were then compiled for all upstream sequence lengths to identify over- and underrepresented TF domains.

### Expression Pattern of CBR Candidate Genes during Regeneration

To characterize CBR genes, we measured their expression levels during the regeneration timeline of a planaria. We used published data sets for *S. mediterranea* from the Sequence Read Archive (SRA) database (https://www.ncbi.nlm.nih.gov/sra; SRA: SRP126428). A heatmap was then generated on Morpheus (https://software.broadinstitute.org/morpheus/) using the associated fragments per kilobase of transcript per million mapped reads (FPKM) values (supplementary table S3, Supplementary Material online).

### Domain Structures for CBR Genes

Domain structures for CBR genes were identified using NCBI Conserved Domain Search (https://www.ncbi.nlm.nih.gov/Structure/cdd/wrpsb.cgi) (supplementary fig. S2, Supplementary Material online). NCBI CDS search did not find any conserved domains for the gene *ARMR*, so we used InterPro search (https://www.ebi.ac.uk/interpro/) to identify any potential domain structures present in the gene (supplementary fig. S2K, Supplementary Material online).

### dsRNA Synthesis

dsRNA was produced as described previously (Rouhana et al. 2013; Shibata and Agata 2018) with a few modifications. Primarily, no plasmid was used in producing the dsRNA. Instead, primers with T7 overhangs were used to directly produce the corresponding PCR template for dsRNA using the Takara PrimeSTAR HS DNA polymerase (Takara Bio, Japan). DNA bands corresponding to the appropriate length were then excised from 1% agarose gels using the Wizard SV Gel and PCR Clean-Up System (Promega, United States). This purified PCR template was used to produce dsRNA using the Megascript T7 kit (Invitrogen, Thermo Fischer Scientific Inc., United States) as per the kit instructions with a few modifications. Briefly, the amount of template used per reaction was increased to 500 ng and the incubation period was set to 20 h. Samples were then subjected to a DNase treatment for 1 h at 37 °C. For annealing, dsRNA samples were incubated at 90 °C for 10 min on a heat block and allowed to cool down to room temperature. dsRNA was precipitated as previously described (Haley et al. 2003; Zhang et al. 2016) by adding 0.1 volume of 3 M sodium acetate (pH 5.2) and 2.5 volume of ice cold 100% ethanol. These samples were incubated at −80 °C overnight to complete the precipitation. dsRNA was then purified with three washes of 70% ethanol, dried, and dissolved in nuclease-free water. dsRNA concentration was measured using NanoDrop One (Thermo Fischer Scientific Inc., United States). All dsRNA primers used in the study are listed in supplementary table S2, Supplementary Material online.

### Planarian Culture Maintenance

*Dugesia* sp. were maintained as previously described with a few changes (Shibata and Agata 2018). Briefly, planaria were maintained in tap water at 21 °C and fed blood worms (Hikari, Kyorin, Japan) every week. All experiments were conducted with planaria starved for 1 week and ∼7 mm in length.

### RNAi Feeding Method

RNAi feeding was conducted as previously described (Shibata and Agata 2018) with a few modifications. Food mixture was prepared using 25 μL of chicken liver solution, 6 μL of 2% Agarose (Sigma-Aldrich, Merck, United States), and 7 μL of dsRNA (∼7 μg/μL). The food mixture was aliquoted to droplets of 6 uL each and frozen at −30 °C until use with feeding experiments. Feedings were conducted in petri dishes housing 20 planaria each and were fed every other day totaling four feeds over a span of 7 d. Roughly, each planaria was fed ∼10 μg of dsRNA. Water was used as negative control, and *DjpiwiB* (Hayashi et al. 2010; Shibata et al. 2016) was used as positive control.

### Quantitative Real-Time PCR Analysis

To measure relative expression levels of CBR genes, we used quantitative PCR (qPCR) by contrasting expression of target genes to the house keeping gene *GADPH* (Umesono et al. 2013) (supplementary fig. S1, Supplementary Material online). RNA was extracted using KingFisher Flex (Thermo Fischer Scientific Inc., United States) and MagMAX Plant RNA Isolation Kit (Applied Biosystems, Thermo Fischer Scientific Inc., United States). cDNA was generated as per the kit instructions in High-Capacity cDNA Reverse Transcription Kit (Applied Biosystems, Thermo Fischer Scientific Inc., United States). Relative expression levels were calculated based on the 2− ΔΔCT method (Livak and Schmittgen 2001). The associated qPCR primers for genes used in the study are shown in supplementary table S1, Supplementary Material online.

## Supplementary Material

evae082_Supplementary_Data

## Data Availability

Genomic data available at NCBI, Ensemble, and Planmine as indicated in Materials and Methods. *S. mediterranea* RNA-Seq data obtained from SRA:SRP126428.
