# Supplementary material for: Conserved Genes in Highly Regenerative Metazoans Are Associated with Planarian Regeneration
Source: Genome Biol Evol. 2024 Apr 23;16(5):evae082. doi: 10.1093/gbe/evae082 (PMC11077316; doi:10.1093/gbe/evae082)
Supplement: evae082_Supplementary_Data [file evae082_supplementary_data.pdf]

Supplementary Tables

Table S1. qPCR primers list for CBR genes and piwiB.

Gene	Strand	Primer Sequence
AVT3C_qPCR	Forward	GCATTTGACAATTTGTACGCAAC
	Reverse	TACCAGTCTGGAATTGCTTTAGG
CAPED_qPCR	Forward	AAGCAGCAAAGAAACGCATAG
	Reverse	ATCGGATCCACATTCTCTTCTG
TIR_qPCR	Forward	TTTGTGGCATCGTGGGTT
	Reverse	GGAATCATCCAAACAAGGTTCATT
AUL36_qPCR	Forward	AATTCCAGCGAGCCAGTTATT
	Reverse	TCCAGTTGATCGTGAGATTTAGC
ARMR_qPCR	Forward	CGCGAATCGCATGTTCTTTC
	Reverse	GGGTGTAAAGGAACCGATCTT
DUF21_qPCR	Forward	GTTAAAGCATCGAGGCTGAGTA
	Reverse	TGGCTATATACGTACTATTGACTCTTG
DISP3_qPCR	Forward	ATTTGTTACCATAACGAGTGC
	Reverse	TTTCCGAATTTGCCTTGATT
MFS_qPCR	Forward	CAATGAGTGTTAGATGAGATTTGGA
	Reverse	TACGCTTCGTCAACCCATAA
HRJDa_qPCR	Forward	CCTTCGGCTCAAGTGGTTT
	Reverse	ATTCATCGTGGCGGCTTT
HRJDb_qPCR	Forward	GGTTGCTGGAGATTGTCTGTA
	Reverse	CATCTGTTGGCACACGAAAC
PiwiB_qPCR	Forward	TGGGACCAACAGAAACGATAAA
	Reverse	CAACAGTCCATGGGTCGTATC
SLR1101_qPCR	Forward	GCGATGTCTATTAGGCTCTGTG
	Reverse	AGTTGTCTTTAACAGGAGGTACAA

Table S2. dsRNA primers list for CBR genes and piwiB.

Gene	Strand	Primer Sequence
AVT3C_dsRNA	Forward	TAATACGACTCACTATAGGGGCATGTGACCAAGAATTCCAAAG
	Reverse	TAATACGACTCACTATAGGGTCCGGTCATTTATTAGAACCAGAAG
CAPED_dsRNA	Forward	TAATACGACTCACTATAGGGTCAACAGAAAGAACGACAGAGG
	Reverse	TAATACGACTCACTATAGGGGTCACACCAACCCAAGGATATT
TLR_dsRNA	Forward	TAATACGACTCACTATAGGGCAATCTGCAATGTGTTGTGAAGA
	Reverse	TAATACGACTCACTATAGGGGATTCTGATTGACGACGCTGTA
AUL36_dsRNA	Forward	TAATACGACTCACTATAGGGACATCTCTTCATTGGGCTTGT
	Reverse	TAATACGACTCACTATAGGGTTCTCGCTTTCGTTCCTTCTC
ARMR_dsRNA	Forward	TAATACGACTCACTATAGGGGTTGAGATGGTTTGATCCTGATTT
	Reverse	TAATACGACTCACTATAGGGGAACACTATCGCCTCTCATCTC
DUF21_dsRNA	Forward	TAATACGACTCACTATAGGGGTCTCCTTCATCGGTTATCTCTG
	Reverse	TAATACGACTCACTATAGGGCTTCATGGACCTACTGGATTGG
DISP3_dsRNA	Forward	TAATACGACTCACTATAGGGACTAAATTCCCAGACAGTTCATTCT
	Reverse	TAATACGACTCACTATAGGGTGCTGAGAACAGTTCCAAGTAAA
MFS_dsRNA	Forward	TAATACGACTCACTATAGGGCAACTCCTGAAATTGGCACTG
	Reverse	TAATACGACTCACTATAGGGGGGAGGAATGATACTACTTGGAAA
HRJDa_dsRNA	Forward	TAATACGACTCACTATAGGGCAAATACCTGTCGGGCATCTAA
	Reverse	TAATACGACTCACTATAGGGGTGTGTTGACGCAAACTGAAT
HRJDb_dsRNA	Forward	TAATACGACTCACTATAGGGTTTGGAACAGGGCCATCA
	Reverse	TAATACGACTCACTATAGGGAGCTATAATGAAATCCACACTTTCC
DjPiwiB_dsRNA	Forward	TAATACGACTCACTATAGGGGACACTACAGAGAGGCACAAAG
	Reverse	TAATACGACTCACTATAGGGAACTCCCTGCCCTCAATTTC
SLR1101_dsRNA	Forward	TAATACGACTCACTATAGGGGGGCTAGCGCTATTCAATCT
	Reverse	TAATACGACTCACTATAGGGGGTACAACAGTCACAGGTACG

Table S3. Fragments Per Kilobase of transcript per Million mapped reads (FPKM) values of CBR genes and SMEDWI.

Gene	0hr	1hr	3hr	6hr	9hr	12hr	24hr	36hr	48hr	60hr	72hr	4d	5d	6d	7d	10d	14d
**SMEDWI1**	127.98	142.73	150.50	141.58	162.07	163.42	227.84	310.53	333.99	346.37	358.74	299.75	276.96	248.15	242.67	207.22	175.67
**SMEDWI2**	121.33	129.47	136.09	135.87	143.88	147.01	185.04	219.53	237.71	257.76	276.43	266.05	263.25	248.52	244.59	216.87	190.56
**SMEDWI3**	126.48	135.88	145.71	145.99	156.97	162.45	204.07	245.96	267.08	283.62	296.63	276.36	265.77	243.95	241.41	210.99	184.45
**HRJDa**	5.57	5.53	6.12	5.49	5.48	4.97	5.66	6.62	6.64	6.92	6.87	7.20	8.44	8.14	8.10	8.25	7.98
**HRJDb**	4.49	2.91	3.20	3.11	3.73	4.48	3.16	2.68	2.48	3.08	4.28	5.26	9.92	14.75	14.70	15.48	12.75
**TRAF4**	1.85	2.27	2.12	2.01	2.30	1.90	2.50	3.45	3.59	3.94	3.80	4.37	4.87	5.48	5.89	6.67	6.82
**CLGIC**	1.39	1.00	1.17	0.89	1.31	0.90	0.71	0.78	0.82	1.07	0.90	1.63	2.08	2.57	2.53	3.00	2.94
**CAPED**	5.38	5.35	5.89	5.25	5.80	5.17	5.01	5.59	4.99	5.04	4.93	5.89	6.25	6.76	6.77	6.74	7.28
**AUL36**	3.07	4.41	3.92	3.45	3.77	3.85	4.57	5.32	4.51	4.85	4.71	5.41	5.54	5.44	5.40	5.55	6.39
**MFS**	10.88	9.47	10.90	9.00	8.96	9.58	10.21	13.32	11.43	12.84	13.88	14.94	15.60	18.09	19.89	20.64	20.47
**FVWA**	0.71	0.65	0.56	0.58	0.82	0.89	0.92	1.02	0.83	0.97	0.82	1.08	1.11	1.18	1.24	1.49	1.60
**ARMR**	4.57	5.57	5.29	4.37	5.54	5.59	6.68	8.52	8.48	7.32	5.64	6.07	6.43	6.97	7.08	6.40	7.49
**FAM166B**	28.56	30.20	30.45	30.90	26.41	26.58	30.71	30.31	24.66	24.23	30.79	26.04	34.19	37.43	35.76	34.77	44.02
**TLR**	7.58	8.98	7.58	6.80	9.05	10.91	11.06	15.16	14.41	12.13	10.24	10.27	11.87	11.32	11.22	10.71	11.58
**AVT3C**	9.26	8.87	9.83	9.37	6.97	7.44	6.41	7.07	7.32	7.39	8.02	7.77	8.96	8.49	9.66	9.98	9.74
**DUF21**	31.80	31.59	35.14	33.20	29.19	31.42	29.56	27.82	24.94	24.62	26.87	23.32	24.03	25.70	21.99	23.93	26.47
**DISP3**	1.23	1.49	1.56	1.20	1.32	1.10	0.98	1.12	1.44	1.53	1.51	1.67	1.65	1.80	1.48	1.56	1.48
**SLR1101**	89.86	94.01	98.90	109.66	106.55	117.20	141.82	153.05	144.50	138.10	133.35	123.57	116.93	115.66	105.31	93.94	93.29

Table S4. First screening for orthologous groups conserved in WBR species.

Nematostella vectensis	*Hydra vulgaris*	*Schmidtea mediterranea*
XP_001623092.2	LOC100201369	SMEST055506001.1
XP_001630801.3	LOC100207979	SMEST008562001.1
XP_001635866.2	LOC100208900	SMEST009387001.1
XP_001638139.1	LOC100213118	SMEST028748001.1
XP_001639440.2	LOC100199666	SMEST010287001.1
XP_001640329.2	LOC100210391	SMEST022950002.1
XP_001640802.1	LOC100205821	SMEST069725001.1
XP_001641095.3	LOC100214193	SMEST078515007.1
XP_032219569.1	LOC100205658	SMEST053118004.1
XP_032228173.1	LOC100206491	SMEST016362001.1
XP_032233355.1	LOC100201023	SMEST077763001.1
XP_032236005.2	LOC105845460	SMEST021796001.1
XP_032240876.2	LOC105845029	SMEST041908003.1
XP_032240898.1	LOC100214466	SMEST033958001.1
XP_032242002.2	LOC100208897	SMEST002733001.1
XP_032242310.2	LOC101241294	SMEST020576001.1
XP_048576621.1	LOC101237868	SMEST021070002.1
XP_048577994.1	LOC124806797	SMEST077977007.1
XP_048578692.1	LOC100206613	SMEST013396001.1
XP_048578947.1	LOC100204497	SMEST058704001.1
XP_048579528.1	LOC100205851	SMEST079952001.1
XP_048583512.1	LOC100204612	SMEST032462005.1
XP_048585397.1	LOC100214450	SMEST029259003.1
XP_048587638.1	LOC100192295	SMEST030353001.1
XP_001637093.3	LOC101237114	SMEST047638001.1

Supplementary Figures

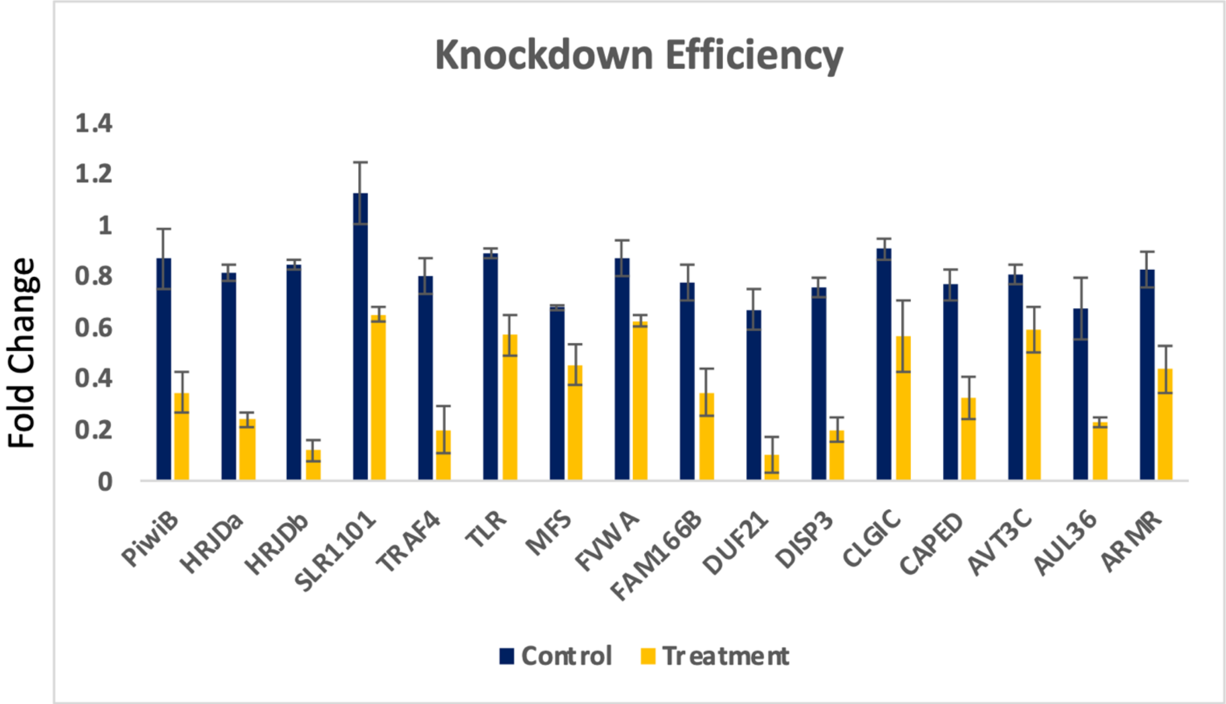

Figure S1. Relative expression levels of CBR genes' mRNA after RNA interference.

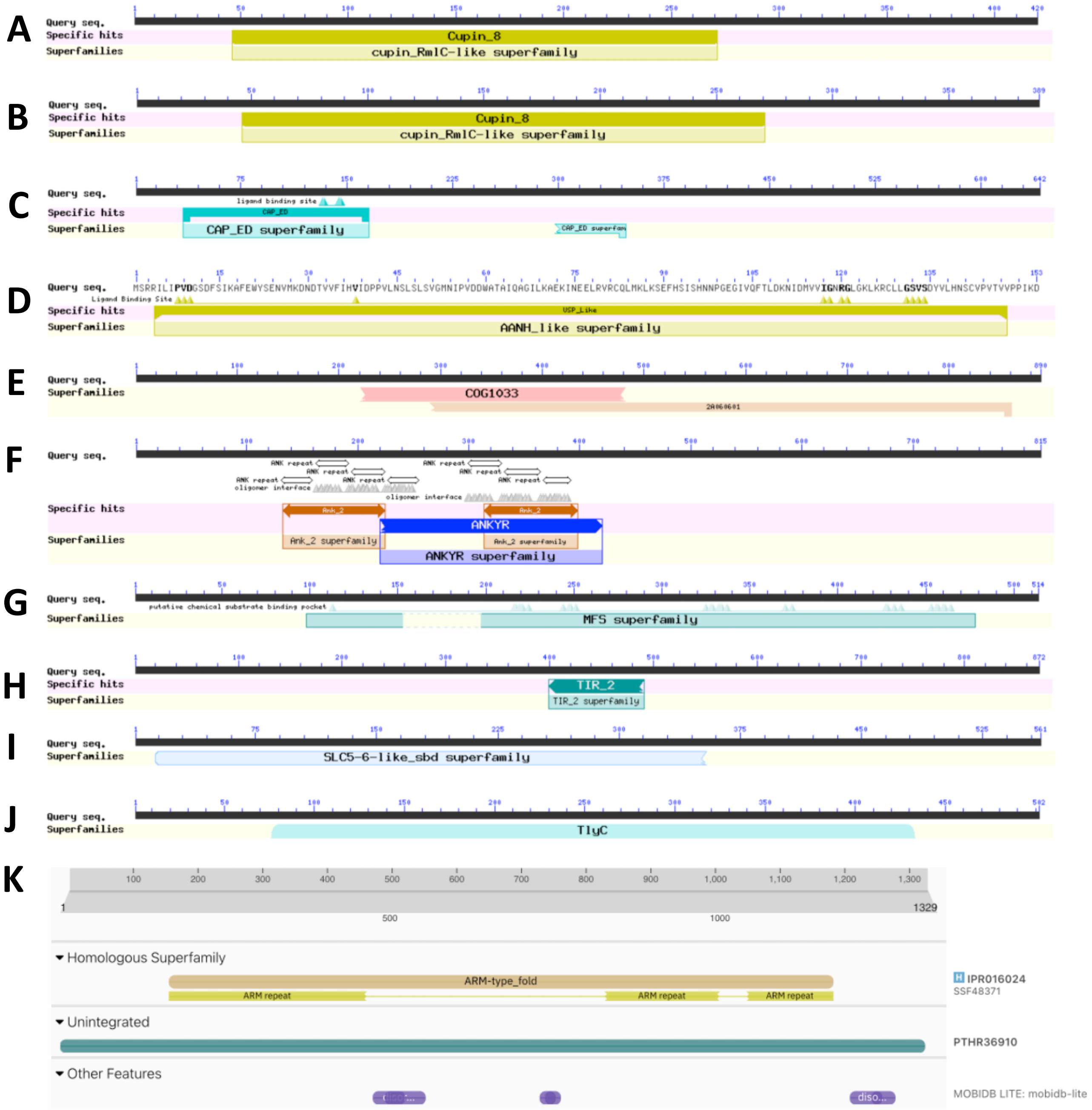

Figure S2. Domain structures of potential epigenetic factors. (A), (B), (C), (D), (E), (F), (G), (H), (I), (J) and (K) correspond to HRJDa, HRJDb, CAPED, SLR1101, DISP3, AUL36, MFS, TLR, AVT3C, DUF21, and ARMR respectively.

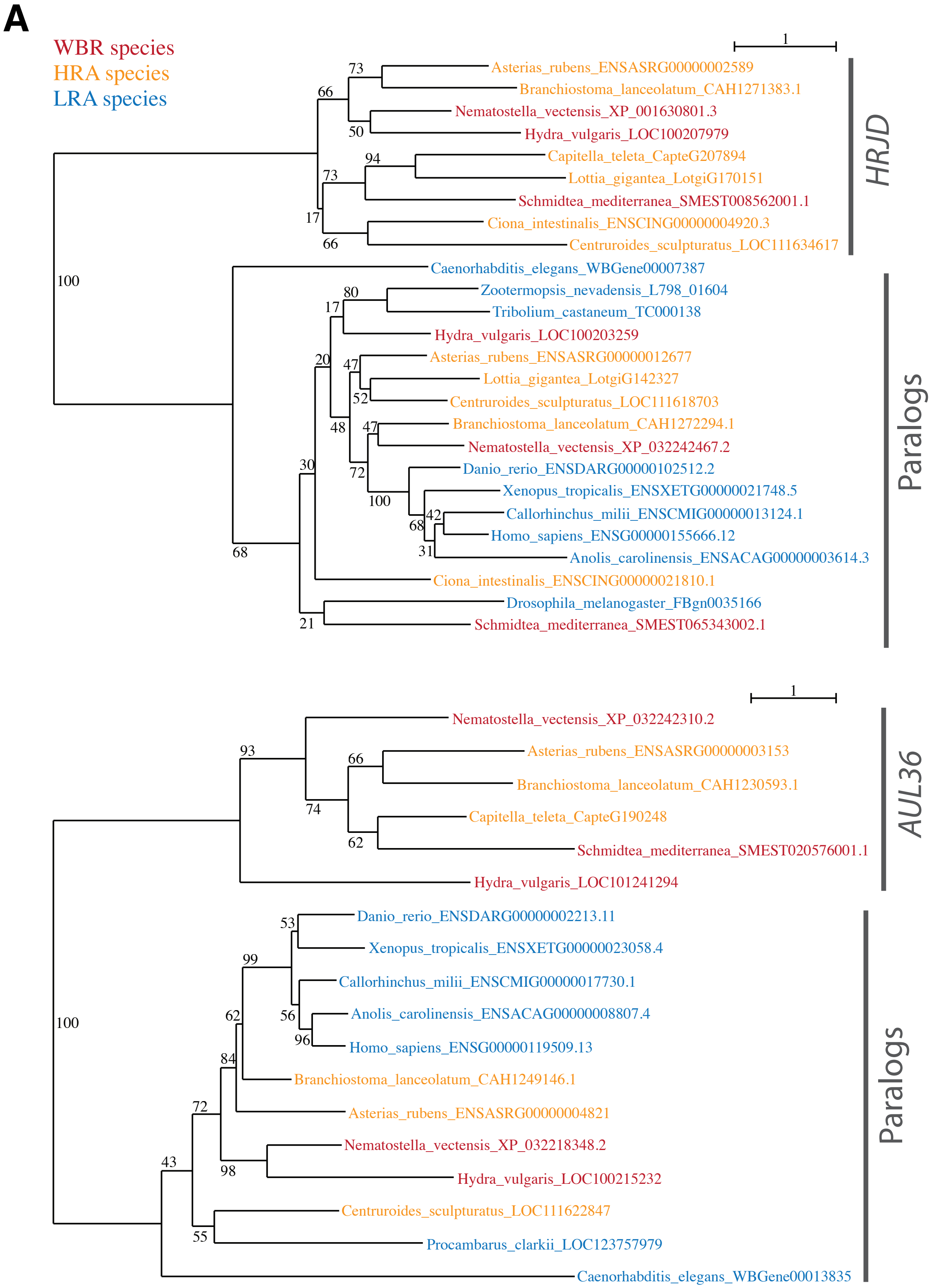

A
WBR species
HRA species
LRA species
1
Asterias_rubens_ENSASRG00000002589
Branchiostoma_lanceolatum_CAH1271383.1
Nematostella_vectensis_XP_001630801.3
Hydra_vulgaris_LOC100207979
Capitella_teleta_CapteG207894
Lottia_gigantea_LotgiG170151
Schmidtea_mediterranea_SMEST008562001.1
Ciona_intestinalis_ENSCING00000004920.3
Centruroides_sculpturatus_LOC111634617
HRJD
Caenorhabditis_elegans_WBGene00007387
Zootermopsis_nevadensis_L798_01604
Tribolium_castaneum_TC000138
Hydra_vulgaris_LOC100203259
Asterias_rubens_ENSASRG00000012677
Lottia_gigantea_LotgiG142327
Centruroides_sculpturatus_LOC111618703
Branchiostoma_lanceolatum_CAH1272294.1
Nematostella_vectensis_XP_032242467.2
Danio_rerio_ENSDARG00000102512.2
Xenopus_tropicalis_ENSXETG00000021748.5
Callorhinchus_milii_ENSCMIG00000013124.1
Homo_sapiens_ENSG00000155666.12
Anolis_carolinensis_ENSACAG00000003614.3
Ciona_intestinalis_ENSCING00000021810.1
Drosophila_melanogaster_FBgn0035166
Schmidtea_mediterranea_SMEST065343002.1
Paralogs
1
Nematostella_vectensis_XP_032242310.2
Asterias_rubens_ENSASRG00000003153
Branchiostoma_lanceolatum_CAH1230593.1
Capitella_teleta_CapteG190248
Schmidtea_mediterranea_SMEST020576001.1
Hydra_vulgaris_LOC101241294
AUL36
Danio_rerio_ENSDARG00000002213.11
Xenopus_tropicalis_ENSXETG00000023058.4
Callorhinchus_milii_ENSCMIG00000017730.1
Anolis_carolinensis_ENSACAG00000008807.4
Homo_sapiens_ENSG00000119509.13
Branchiostoma_lanceolatum_CAH1249146.1
Asterias_rubens_ENSASRG00000004821
Nematostella_vectensis_XP_032218348.2
Hydra_vulgaris_LOC100215232
Centruroides_sculpturatus_LOC111622847
Procambarus_clarkii_LOC123757979
Caenorhabditis_elegans_WBGene00013835
Paralogs

B

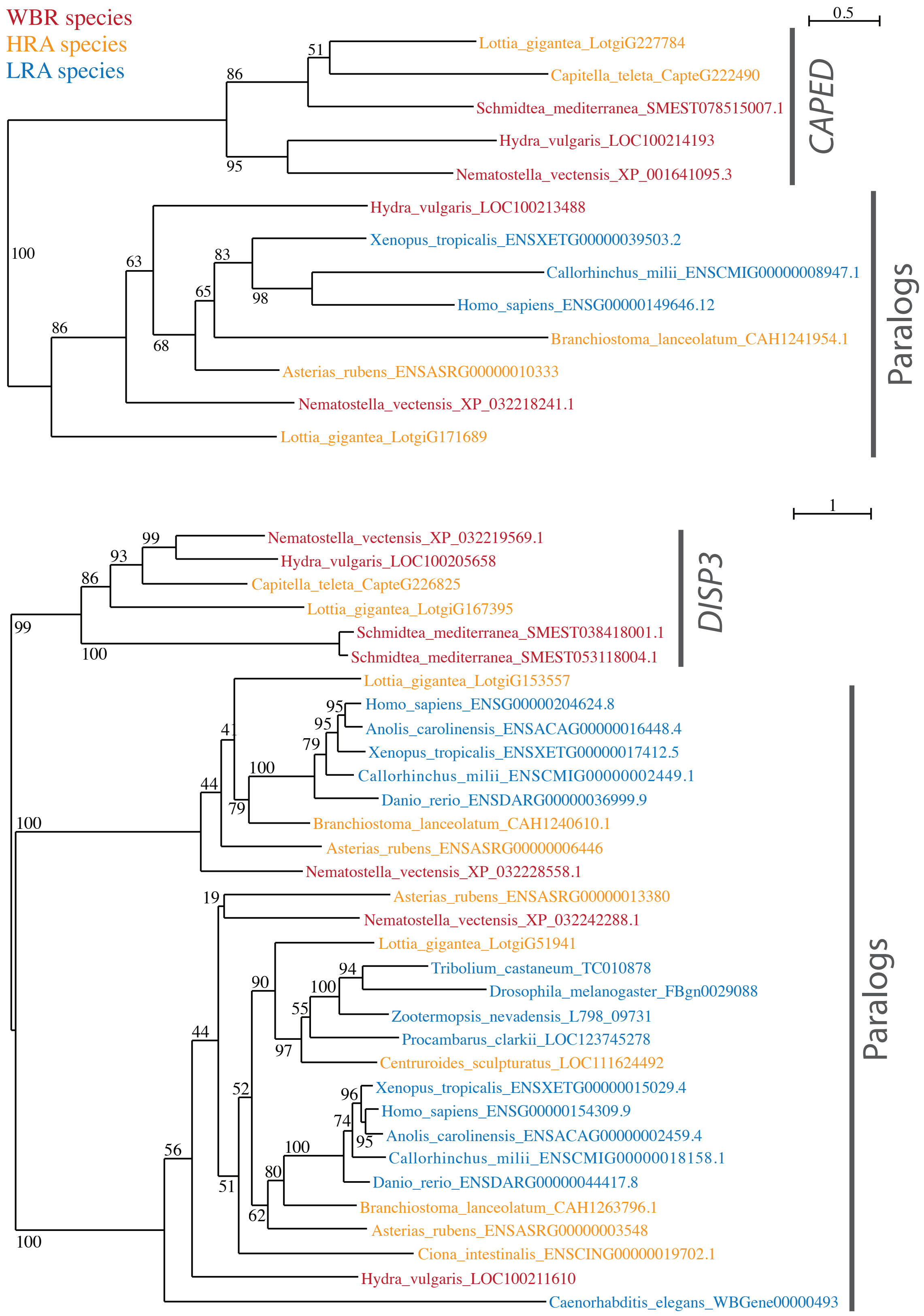

WBR species
HRA species
LRA species
0.5
CAPED
Paralogs
Lottia_gigantea_LotgiG227784
Capitella_teleta_CapteG222490
Schmidtea_mediterranea_SMEST078515007.1
Hydra_vulgaris_LOC100214193
Nematostella_vectensis_XP_001641095.3
Hydra_vulgaris_LOC100213488
Xenopus_tropicalis_ENSXETG00000039503.2
Callorhinchus_milii_ENSCMIG00000008947.1
Homo_sapiens_ENSG00000149646.12
Branchiostoma_lanceolatum_CAH1241954.1
Asterias_rubens_ENSASRG00000010333
Nematostella_vectensis_XP_032218241.1
Lottia_gigantea_LotgiG171689
1
DISP3
Paralogs
Nematostella_vectensis_XP_032219569.1
Hydra_vulgaris_LOC100205658
Capitella_teleta_CapteG226825
Lottia_gigantea_LotgiG167395
Schmidtea_mediterranea_SMEST038418001.1
Schmidtea_mediterranea_SMEST053118004.1
Lottia_gigantea_LotgiG153557
Homo_sapiens_ENSG00000204624.8
Anolis_carolinensis_ENSACAG00000016448.4
Xenopus_tropicalis_ENSXETG00000017412.5
Callorhinchus_milii_ENSCMIG00000002449.1
Danio_rerio_ENSDARG00000036999.9
Branchiostoma_lanceolatum_CAH1240610.1
Asterias_rubens_ENSASRG00000006446
Nematostella_vectensis_XP_032228558.1
Asterias_rubens_ENSASRG00000013380
Nematostella_vectensis_XP_032242288.1
Lottia_gigantea_LotgiG51941
Tribolium_castaneum_TC010878
Drosophila_melanogaster_FBgn0029088
Zootermopsis_nevadensis_L798_09731
Procambarus_clarkii_LOC123745278
Centruroides_sculpturatus_LOC111624492
Xenopus_tropicalis_ENSXETG00000015029.4
Homo_sapiens_ENSG00000154309.9
Anolis_carolinensis_ENSACAG00000002459.4
Callorhinchus_milii_ENSCMIG00000018158.1
Danio_rerio_ENSDARG00000044417.8
Branchiostoma_lanceolatum_CAH1263796.1
Asterias_rubens_ENSASRG00000003548
Ciona_intestinalis_ENSCING00000019702.1
Hydra_vulgaris_LOC100211610
Caenorhabditis_elegans_WBGene00000493

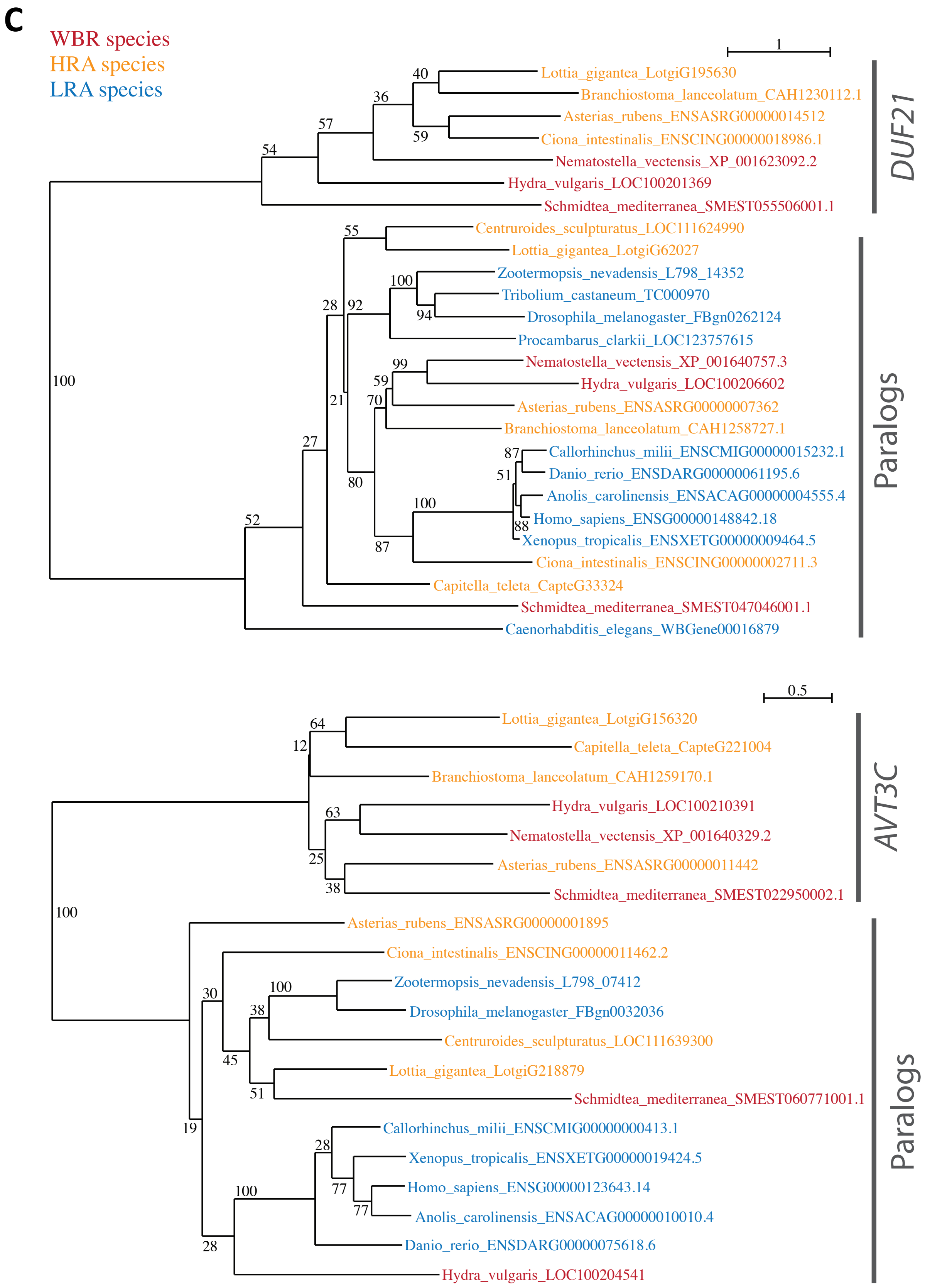
C
WBR species
HRA species
LRA species
1
Lottia_gigantea_LotgiG195630
Branchiostoma_lanceolatum_CAH1230112.1
Asterias_rubens_ENSASRG00000014512
Ciona_intestinalis_ENSCING00000018986.1
Nematostella_vectensis_XP_001623092.2
Hydra_vulgaris_LOC100201369
Schmidtea_mediterranea_SMEST055506001.1
DUF21
Centruroides_sculpturatus_LOC111624990
Lottia_gigantea_LotgiG62027
Zootermopsis_nevadensis_L798_14352
Tribolium_castaneum_TC000970
Drosophila_melanogaster_FBgn0262124
Procambarus_clarkii_LOC123757615
Nematostella_vectensis_XP_001640757.3
Hydra_vulgaris_LOC100206602
Asterias_rubens_ENSASRG00000007362
Branchiostoma_lanceolatum_CAH1258727.1
Callorhinchus_milii_ENSCMIG00000015232.1
Danio_rerio_ENSDARG00000061195.6
Anolis_carolinensis_ENSACAG00000004555.4
Homo_sapiens_ENSG00000148842.18
Xenopus_tropicalis_ENSXETG00000009464.5
Ciona_intestinalis_ENSCING00000002711.3
Capitella_teleta_CapteG33324
Schmidtea_mediterranea_SMEST047046001.1
Caenorhabditis_elegans_WBGene00016879
Paralogs
0.5
Lottia_gigantea_LotgiG156320
Capitella_teleta_CapteG221004
Branchiostoma_lanceolatum_CAH1259170.1
Hydra_vulgaris_LOC100210391
Nematostella_vectensis_XP_001640329.2
Asterias_rubens_ENSASRG00000011442
Schmidtea_mediterranea_SMEST022950002.1
AVT3C
Asterias_rubens_ENSASRG00000001895
Ciona_intestinalis_ENSCING00000011462.2
Zootermopsis_nevadensis_L798_07412
Drosophila_melanogaster_FBgn0032036
Centruroides_sculpturatus_LOC111639300
Lottia_gigantea_LotgiG218879
Schmidtea_mediterranea_SMEST060771001.1
Callorhinchus_milii_ENSCMIG00000000413.1
Xenopus_tropicalis_ENSXETG00000019424.5
Homo_sapiens_ENSG00000123643.14
Anolis_carolinensis_ENSACAG00000010010.4
Danio_rerio_ENSDARG00000075618.6
Hydra_vulgaris_LOC100204541
Paralogs

Figure S3. Phylogenetic relationships of CBR genes. (A-C) Phylogenetic relations of *HRJD* & *AUL36*, *CAPED* & *DISP3*, and *DUF21* & *AVT3C* with their paralogs respectively. Trees were built using the maximum-likelihood algorithm of RAxML. Bootstrapping was performed with 100 replicates and bootstrap values were calculated for each node as indicated in the figure.

Supplementary Data: Amino acid sequences of CBR genes in *Schmidtea mediterranea*

>DUF21, SMEST055506001.1 gene=SMEST055506001.1 seq_id=smed_chr1 type=cds
MIEDIWCTQINAEQIICNGTEFIKYEPPLDIFDYYFWVYLGIYILLILGAGLMSGLTLGLLSL
DALTLEILMRAGAEPEAKYAKKLYPIIKRHHILLVTLLLGNAACVESMPLFLDRISNPITAVI
VSVTAVLIFGEIIPQAFCKRYGLAIGFYMRPVVYFMMGITLVVGYPISKLLDCIFGHDNSN
YYRRPQLKALIDLHGPTGLIVKSYERKSYRNSRQFENDEEESKVINNSNDHHLSIEEVNII
KGALDLTTMTAKDALVPLDHVFMLSDKAILDKQTIHQILLVAHSRIPVFMGSERRLIQGIL
LTKMLIQFNSSENIKIEQLIHNNKCFQPLRYVTETTPLYDLLQLFKTGKTGHMAVVVKNNI
ADEQLDALERFYSIGNKISSNLVSVFPPVIGIITLEDVLEKLIQSEITDEGDIFRHDICRRLIY
KQKLRWAKFKVTKGMTFENEFKNKRIGDSEIINEYVSNNFEPTNKDFEDQTHLLSSTQPQ
IYRTFLNV
--
>DISP3, SMEST053118004.1 gene=SMEST053118004.1 seq_id=smed_chr1 type=cds
MSNDGEEIVVNDYISSKEELDFRYKSFFFFLPLSLQPRPYQCYKTWEYIDGLSFKYGFDFSI
AKDYGLRSETQEFRFYYDSVDGNILTPSNLRAINEIYNKYLNLFDKIKSCMGPCLRYPTV
VDVLLGNNGNDSTFPNLDQTIDKVLASNYKAMFQRALSKDFAKKNGKYVARCIKSETII
AYIGLGVIREFMNEYYNIYNEYYSRLYLYIEYKYLLGELLLDTIFKDIYLTLGSLVYVYFFT
YIQTKSFFITFFEIFSIFTTFCISNFIHTVILGIKFFGIFHVLSIFLILGLGCDNLYVFYDRWKSF
ENINFISLTHRLYKTYIGVGWSMFITSVTTALAFITLAWTKLLTIFSFGVFVALLVIVNYISLL
LYLPSVIVIYHNKFHNVLKPDKIFIFVTKFFKVDYFNFLKYKSVKYVILVIFGVLTIGGFICV
TQIKVTEEFPSLFVSRFSYSRMEYLKSNYFAREHVDVNLQIVLLFGIKRLDYNACEYWNY
TCRGTTVFDQKFSINSPGIQEEYKNICDTLITADKNLKTELSLFDGLEHINCLLYNMNDYY
LPRFPTKNFQIPWKTSNFMELVSVMGPNFTAGPDFPYKFETALYYYLTDGFTFSDYGKYYI
FRINRKIQRTKPMRYGLLKGYSLNLSAILVNITLFQKLSGFSEKKKVFNSWIKWTNKLNK
QFHVMVCGSQSDISLNGYFHWIHVQELLIGSAVAGILSGLIISCPIVWFATLNVHTFLIILIG
TCLSTLSVTSLIYITGWNLGLLESINLTVVVGLTIDYLVHMGLAYNSSQKKHRDERTEDML
EEMGVSILSGMSTTLGSSFFLFFCQTKFLFQFGVFVFATIFSAGIYSMIFLPCMFLVFGPSQG
RGDFHCDFYRCMPCLKTEKRSEKYK
--
>HRJDa, SMEST008562001.1 gene=SMEST008562001.1 seq_id=smed_chr1 type=cds
MPQSSTIIFIILYFINVNYSFHVNHNKPFGTGPSVSIDFTNGFVDPITFFESFVQKNKPLLMK
QAAKNFPAMEKWDDEYFLNLNLSKYYPNTVNIETKKKENRTLKNMEMNFKDFIKSYND
SDIYMVNNLPNFLNEDIHFPWPLQCDLFDENIFQVIMWFSSGNTSSVVHTDDMENINCLI
RGTKTFVLVDPEKHGDKIPMKKEGAYSDIDVDRVDFSKYKKMENVDFIIAEMEAGDCLY
IPKLWIHQVRSFHRNIAINIWFRVPEMFFEISNKTELLEKCNKYPDDKKTFSNTNYLGLNT
GIYNRNDGIEMLSELQNKSVSSAFVTILEIYNDIDLKILMKFSDHIKLVIKEIIDEVDQNKNS
IIDEEEIKLANSKNLSRIANYINYLKTFLESLSIKEEIKNLVDTSETNNLNQHNEL
--
>HRJDb, SMEST008562002.1 gene=SMEST008562002.1 seq_id=smed_chr1 type=cds
MPQSSTIIFIILYFINVNYSFHVNHNKPFGTGPSVSIDFTNGFVDPITFFESFVQKNKPLLMK
QAAKNFPAMEKWDDEYFLNLNLSKYYPNTVNIETKKKENRTLKNMEMNFKDFIKSYND
SDIYMVNNLPNFLNEDIHFPWPLQCDLFDENIFQVIMWFSSGNTSSVVHTDDMENINCLI
RGTKTFVLVDPEKHGDKIPMKKEGAYSDIDVDRVDFSKYKKMENVDFIIAEMEAGDCLY
IPKLWIHQVRSFHRNIAINIWFRVPEMFFEISNKTELLEKCNKYPDDKKTFSNTNYLGLNT
GIYNRNDGIEMLSELQNKSVSSAFVTILEIYNDIDLKILMKFSDHIKLVIKEIIDEVDQNKNS
IIDEEEIKLANSKTFHQFYEQQYY

--
>CAPED, SMEST078515007.1 gene=SMEST078515007.1 seq_id=smed_chr2 type=cds
MYEMVVSCITKPSKDRTPDEIISISPWLRKKAALFDSLKEEVLNEIIKSCSFSERRRDDIIIR
QGDKGDCFYVMLAGRVSIFINTEAVPDNPEDANVVVTEDKFKDEGMAKKELDRSRYGN
FIAILDSGKAFGELALINTDCVRNASIIADELTHLLVVNRELYNRSIKEIQTQEFNERLSFVQ
NSALFTNWLSKYKRQVAFSLRKEKFGFNMNLITQGEPTNGLTFILNGHAKVVVNANQHV
SQYPWLNATINNEELRLSDVNDLDKSVDFETKKPLHNSLSKRLPYIEAEQRQAQRNIEVCI
VKGGEIIGDIEQGLELTTYASSVITIEATEVYILDAKNFERLCNPKKNPKTLNLLKKKAIYKI
KHRGNRLEFRPIPFYIRLNEMIADEEDRETRESDLKNQKIEALKNNEKGDFNFFQHRGPLI
DLYGPGSVFYRNRQLRKEKQIIERKRQITEMALKGRRGNQRGGVSIGMFGNEKFENSDD
DSEDGEEINTISHFNNYNNNQNVDCIDYLKGFNAHDSMNDWETGSVHLQSLEDRLRNW
FLSVSSLTNDKRAGRAIEHYTILKRFKQEDLEKKIIPGKKIFMRPKKQTIKHMNLTDDNRN
EVFITAPKLKTTPTNLKKTFEDAVESNSESLYHSTK
--
>SLR1101, SMEST079952001.1 gene=SMEST079952001.1 seq_id=smed_chr2 type=cds
MSRRILIPVDGSDFSIKAFEWYSENVMKDNDTVVFIHVIDPPVLNSLSLSVGMNIPVDDW
ATAIQAGILKAEKINEELRVRCQLMKLKSEFHSISHNNPGEGIVQFTLDKNIDMVVIGNRG
LGKLKRCLLGSVSDYVLHNSCVPVTVVPPIKD
--
>AUL36, SMEST020576001.1 gene=SMEST020576001.1 seq_id=smed_chr2 type=cds
MTVKRRDFQKIATTIHQFLLKLTDTYECAELRDSLNMSLLQLQKYYRRCQQRLQHTGRH
NDQNGTGYVSPRQSLRITLSRSSEALSPMTPYIEEPQYFHAHWTVLDEKSDQIRKSFSQEN
FSPGRSINTPPVHYAATKGDFELLNELLLQKKDVLEDQDCLGRTPLMYAVYYDKIEVVRW
LLKRGADPNSCDLSEITSLHWACFKGYVALIELLLDAGANINTQDSFGRSACHWACTYNT
VEPLQLLIEFETKVNQHDRNGLTPAMWACRLDHLSHLNVLANVKNQEIDHIDGFERDRN
GRTIVHWSIRRQLPLECLKYLVNEHTVQLKDDEGKTVLLLAAEQGALETCKVIIEKFGSK
YIEDVDDQDRTSLILATNGGHGEVVNFLLENGANIKFKDRNGASCVDIARNQYLFYCQLI
ISSHIREMERRQESIPSDCVELIEKANSRHNSISEARQQVKTPPRPPVSPKSPSSQSSKSPTIK
RLPIKYARTKNIDNDETPNGFDAMSSDPYQYPFNDLGTKTDSNDNDIGNHHENPLNSTEL
SRTLENFQYQRPQTLDVLDNETGQQYHRSYEHLHSNRSADNSRSENLENKLPSPIQIETH
KPIQRAKKVNRINGNNVNMPHFTPPELHPLTSETSENSQSVPAVNNPVKHIRSNSHQLSIM
EISQKVNYGASLEPINKIHSNETPVRKKKKKSKDQRILAKSQDRLGNLNFDQDQNSKSLG
TMQHARSLENMPNKVKQLPNGIISNRNSSFSETFPSPYGEQSIKGNRQNSLLFDDRQRFEQ
EALTDKPNGKNLSSFEIRSSNWLKNGISQF
--
>MFS, SMEST058704001.1 gene=SMEST058704001.1 seq_id=smed_chr2 type=cds
MYKKLSVFDDNEELGDNCINSIIQRQANMDADYNTNLISSDQEDEDLDVPKVHLSLFKIII
LNMTYLGLSMMFLFLSVVVVPAQIEIIVGSQYKGIVLGITVGIGAIFTFVIGPLVGMKSDRL
VSTYGRRRPIMVISVISLCISCIIMVAAAPSINVHVYNKNISNSTECIRPNLVEQRCANPNILI
NNKLVPLNVSYVNRLWLYIPLYLVVIISYVSTITPYNALIADKSHPDQRGLSSGAMGGMIL
LGNIFGASLGLLITSLGAVFTVCIGIVLLLVCVTITVSLTKEYETVHKPIPMSCGEKFKMFW
LPLKDHDFRWLFITRFLMQQGLTSVIGFLEYWIKDMIDLPLCWNTYKGVSYLLLPMLISA
SISSVLSGIASDKLGRRKIILTINSFIMAIMVVLMACLKGEKAYYLAIFLAIIFGVALGGYQS
VDFALLMDIIPDEKDQSLMIAVWHQAMILPQALAVPVSGVIVDLFERVNCQIGLGYIILLL
LIAVYFFLSGCFIRKIELST
--
>ARMR, SMEST021070002.1 gene=SMEST021070002.1 seq_id=smed_chr2 type=cds

MNQLYDTIEMCFDISNTNDECDFLQNFISCVISDNEESDNDLMESKDKLFYKEVLYNDSKI
RCNFENIYNCSKTTGVNWKVAEDLDWFLFYVFYRRFGWPDLEEENGMLKVVLPDSQIR
FRKSSKSEELKYKPQLVDMLDSIILWLSHQPISAVSMLMYSSAIRLASKSPVTRLHLSIRGY
DWLKENLTRDINDKLWHTLQEFLILGITDFWSRIRDSAILRLGPIIERLELSQIFSLFDELIRL
CDSNVNQWQIVDGAISAICAILKRFQKVGYKSEIIPSKIYSSISSPRFHCQIKIGDNEIDRMP
EHIMSKIQFLIFKLIYHPQQTIRDNSIKAFVLCLDRYYENDVDTAFQNVCQKLSCRPNGSP
AFNNSNSNIKSLPNQYQRYIDPYGSQSLICLSHHLFKRIHDKINYLENWHHYHCYFDQYL
SHPASSVRLVSSNLYTYVVSQAISHPLLVKNIIHNLIIKWSPDQNVLLKSISSLIKSLDSKHR
KRISVSKIESDNDVSEAWEWREGRLLVFELFAKLIIKNHLWYTFGTTEVESVYSSDNTNSR
LNESSLYDAVLDHPITLSGSSIGINNEQSLDDTEDSRLSKSFDARRTTITSFPIRNIKFKALSV
NSIPPKSKTEDGTKRRKSHDIILRKIGEDESLNKVPVMRKLSISRLNVATSTKNLHSISHSSF
LLHIKEIESFEEAKLSNTKININSQSNNEPKLPTFRSLLSQMLHTSAESLIDSQWELRRMAK
QALPPLGEVLRWYDPELLVNIINTHLKFEPTLMTAISLQLLHDSLLHTSRLKQLTTKPPSS
WQPNVAMRVVNNIITRIESNLPQWMKSCQHYLDRSTVFDCITPHSINIVITCISNYSDIISAI
NLSKSLVEAFVRLDKDIRCQGSNFISIYSFEIYPNSPSNIISSENPALEYAVQLLDFIKQGLLG
WWQKLANLILIRDYYDLHKEAIISFAKLMTIFACWIYPISEMRGDNARYIRDINKLMGFLL
KLLIEVMGIDVSTRLIFATNLVQLLEVLASYMQEKTIKLDNWWMRQLMELMFLWIEVFD
LNELEFVCRTEIEGKKPILFSTLKALLQRLHSMSNRRRATVGSINVAQMSKSKTITKRHSIC
ENSDSPILNGSNFIRTENANNLELFSSLAIGENLEYISDESLNDSDGEKKTGSYRESHVLSGI
HPNRAHSLQLPLEFGIKKSFTLLPGKSNSSLIYEEADLEKRSFRRSCSSVNSEFNDELEASD
WDSSSESEGNSAFNSPAKADKSNDETAIISELITKLIKYNPKLFEQSKQFLPDFDTTILQGL
KI
--
>TLR, SMEST077763001.1 gene=SMEST077763001.1 seq_id=smed_chr3 type=cds
MELLDEINFQINGKLRKIELYLGDITKLKLIDKVDLLLVSSFPSNYSPIPGTLIGALLNSLSV
SVKDLAKDKSEDLRKDFHCWISKPLSDKVPYKRILCFEQGKKQNDFGYLENPAALVGAV
FRALFTAFSDDEWSLITPILATGNQGSDKKTMIRAMIKGCSSWIIAGMPLKSMKIVIYRAK
ETSFVGKYDQEAIDEFSKLKSEMNWKPVFDQVIAIKYDLLILFTKQDEDLIEPFMKCFTDL
ISEDNRTIIFQCLDEMEKELDDQKKFNFQQKMIETKKVVVFLTPTFLTLSQAVELYNLALC
LTHKTRRNLLTPIYLKNISQLPEYMSLNQWIDCRIKNSEDLLEIKFVNASQSLWSILKECDD
WKIKNKTQSENGSYSNTLVNSFDQSLYEKFDIFISYCHKDSKIANIILSFLRISLPNIKIFFDV
SGIKSGYSWQETIYLGINNSTIILALLSENYIQSTMCREELSLATSQHQSPKFSSHLIPIWISN
TELKIEQSVNNIWKTFSFVQNILIPKFPNPESTDLNLKYISFCQELLQLYILPILSNEVTRKES
SILPVINLEDAALQARISFFKSTWSMKTFNTHPNQVKGVLCQYPRLPFEIVIAFHEDDLLF
AQILQNFLLIINKGLKIKFSSVDPKGNDYNSFSYFDQANLIIILLNPYYISTKACINQLEIALS
LLRRQSEVGFRVCPLILESLPSQPSYIHLLPSRFKSDDEIYQEILNKHRDKINKIFYCISEGSI
KNKVVTAKAQVMLYFVASWIGESLFSGRYFIAPKTPVIFSTYDLINTNFNDPVFDSSLDIN
DQIEFVLISSESLKLGKRFIVTHDMVFKNVDWVSNSSTSHQLPGKSNEMSVVNVVKTKA
CLLM
--
>AVT3C, SMEST022950002.1 gene=SMEST022950002.1 seq_id=smed_chr4 type=cds
MDQVTTLQKFIDFANIFKAFIGCTYLTIPFAFYQSGIGLGIIMLLIIGWLNDYCCVQIVKCK
DIIVARLIEQFDNKYKSGNLLEPEAELVRCGHLKDKLEDRITLGHIAKYAFGKKGQWIVTI
CLLISQFGFCTGYFVFVGNTLMELIYNLNSNHSDTSLNSSSITYPIFNIKKDHFLIFMLLVPL
PIFILFSLLRTVRNMGWISIIANSAIAFGYFSIVGLLINHFSKSEDFELFNFQSVAVIFGILTAG
FEGIGTVIPIETSMIGNRHNFSRYLHVTLFLVVCVFGSFGILGHLRYGTNTKQIILQNLPYST
FVNIINLILIFSVVCTYPLQMFPVVEIVEFLFFRKRSAEFVEDRIQILSSNAVNEQKSDLETID

DELLNELSGKESDDDDEREVKRKSLRRFKKSVDKPAPNYGAVDNTNASVLFEGTDTNLN
ENMKSTLESVYTKKQLRMIRVLNTTNTSAPTWKRNIIRIILVLLQLSLALVMRNNFAYLSA
IIGAVGSSFLCYILPAATHLKLTRETDGRLYCKILDVIIVIFGIIGSVASLVVTIVQMVRKDF
